# A Comparison of Gaming Behavior between Teens and Preteens and its Association with Depression, Anxiety, Stress, and Academic Performance in Children

**DOI:** 10.2174/0117450179393523251022062235

**Published:** 2025-11-26

**Authors:** Suzie Rababa’h, Karem H. Alzoubi, Rania Mahafdeh, Iman Basheti, Laiali T. Alquraan, Bayan Mahafdeh, Ahmed Alhusban

**Affiliations:** 1 Department of Pharmacy, Faculty of Pharmacy, Jadara University, Irbid, Jordan; 2 Department of Pharmacy Practice and Pharmacotherapeutics, College of Pharmacy, University of Sharjah, Sharjah, UAE; 3 Department of Clinical Pharmacy, Faculty of Pharmacy, Jordan University of Science and Technology, Irbid, Jordan; 4 Department of Biological Sciences, The Faculty of Science, Yarmouk University, Irbid, Jordan; 5 Faculty of Medicine, Yarmouk University, Irbid, Jordan

**Keywords:** Academic performance, video gaming, Teens, Preteens, Depression severity, Anxiety severity, Stress severity, GPA level

## Abstract

**Background:**

Video games have emerged as a significant and timely source of entertainment among teens and preteens, primarily targeting the younger generation while also gaining popularity among the older population. Numerous studies have demonstrated that symptoms of stress, anxiety, and depression can impact children’s academic performance and may result in school dropout. However, to date, no research has examined these three conditions specifically in Jordanian high school students. Therefore, this study aims to compare the gaming behavior of teens and preteens and its relationship to memory, depression, anxiety, and stress in Jordanian schoolchildren. Additionally, it seeks to define the prevalence of anxiety, depression, and stress among high school students in Northern Jordan and identify factors associated with these conditions.

**Methods:**

This cross-sectional study, conducted between May and July 2024, involved a sample of 388 children aged 12 to 17 years, randomly selected from public and private schools. Validated versions of the Arabic versions of the Patient Health Questionnaire-9 (PHQ-9), the seven-item Generalized Anxiety Disorder Scale (GAD-7), the Perceived Stress Scale (PSS), and the Internet Gaming Disorder Scale–Short-Form (IGDS9-SF) were used to assess depression, anxiety, perceived stress, and internet gaming disorder, respectively.

**Results:**

Among the 388 students included in the study, 25 students (6.44%) reported using gaming apps during classes on a frequent and daily basis. The findings reported that gaming during class was significantly linked to higher depression scores (p < 0.0001). Additionally, students with high GPAs were more likely to experience severe depression (p = 0.02), suggesting that academic pressure contributes to mental health struggles. Anxiety was a strong predictor of depression severity, while excessive gaming also emerged as a significant factor in both mild and severe depression. Older students had higher intermediate GPAs, suggesting that maturity and time management skills may positively impact academic performance. These findings highlighted the complex relationship between gaming, academic performance, and mental health, emphasizing the need for responsible gaming habits and mental health support in educational settings.

**Discussion:**

A comparison of gaming behavior between teens and preteens indicates that teens exhibit higher levels of depression, anxiety, and gaming habits than preteens. This study highlights the complex interplay between gaming behavior and academic performance. While gaming itself does not directly lower GPA, it may contribute to psychological health issues. Additionally, high-achieving students may be at greater risk of depression, emphasizing the need for mental health support programs in educational settings.

**Conclusion:**

Current findings stress the importance of responsible gaming habits, early psychological interventions, and targeted mental health strategies for students.

## 1 INTRODUCTION

Video games play an integral part in every aspect of children's daily lives [1-3]. Between 2011 and 2017, the amount of time spent playing video games rose from 5.1 hours per week to 6.5 hours per week [4]. While moderate video gaming may offer certain cognitive benefits, such as improved working memory, multitasking, and attention [5, 6], excessive video gaming can be linked to poorer academic performance, peer conflicts, and diminished social skills [7-9]. In recognition of these risks, the World Health Organization (WHO) classified gaming disorder as an addictive behavioral disorder [4, 10].

Previous studies investigated the relationship between excessive gaming behavior and mental illnesses like stress and revealed that stress can affect academic performance [11-14]. A significant body of research demonstrated that the onset of mental illnesses commonly starts during childhood and adolescence [15, 16]. The prevalence of mental problems in children and adolescents ranges from 1% to 51%, with a mean rate of 15.8% for teenagers [17]. Depression alone accounts for 4.4% of total disability-adjusted life years globally, making it the fourth leading cause of illness burden according to WHO reports [18].

Recent studies have highlighted a significant rise in such mental health issues, particularly following the COVID-19 pandemic [19]. Choi *et al.* (2023) analyzed the changes in prolonged trends of sadness, counseling, and sleep time among adults before and after the COVID-19 pandemic [20]. They demonstrated a noticeable increase in the prevalence of sadness, a decline in the average sleep time, and an increase in the prevalence of depression during the COVID-19 pandemic among Korean adults [20]. Such findings suggest that broader societal events may have contributed to elevated rates of mental health issues observed in recent years.

Mental health complications, such as anxiety and depression, affect not only individuals but also extend their impact to surrounding social networks, reshaping family dynamics and straining interpersonal relationships [21, 22]. Moreover, these conditions are associated with substantial economic costs, due to both direct medical expenses and indirect costs, such as productivity loss [23-25]. In light of these trends, preventing mental health complications has become a significant goal of public health strategies [26, 27]. A recent systematic umbrella review included seven meta-analyses of prospective cohort studies, demonstrating that physical activity significantly decreases the risk of developing mental health disorders [28]. These findings underscore the importance of promoting physical activity as a preventative strategy for mental health complications among adolescents. However, there are other factors, such as lifestyle habits, socioeconomic status, environment, genetics, and pre-existing health conditions, which may independently influence mental health outcomes [29-31].

Despite the published research discussing the relationship between gaming and mental health, the vast majority of studies have been conducted in Western contexts. There is limited data available on Middle Eastern populations, particularly Jordanian students, where their cultural, educational, and digital habits may differ [32]. The main aim of this study is to investigate how gaming behavior among teens and preteens is related to academic achievement, stress, anxiety, and depression in Jordan.

## 2 METHODS

### 2.1 Design

A cross-sectional study was conducted between May and July 2024, involving children enrolled in schools between the ages of 12 and 17 years. Five schools (private and public) were randomly selected for this study. Students were randomly chosen from a list provided by the school administration. The questionnaire, along with a brief description of the survey, an assurance of data protection, and consent, was shared with the parents of the pupils. The Institutional Review Board of Jadara University in Irbid, Jordan, approved the study (approval number: PHARM-JA-5/2024).

Written informed consent was obtained from all parents and students prior to participation. Students were also provided with an age-appropriate explanation of the study’s objectives, and verbal consent was obtained from each child before data collection. We also mentioned that participants were informed that their responses would remain anonymous and that participation was entirely voluntary. Students were told they could skip any question or withdraw at any point without penalty. Additionally, participants were provided with the contact information of the school counselor and encouraged to reach out if they felt distress or discomfort after completing the questionnaire.

We used G-Power software version 3.1.9.7 to calculate the sample size. A significance level of 0.05, a power of 0.90, and a medium effect size of 0.30 required the minimum number of subjects to be 342. Based on an anticipated dropout rate of 15%, the target number of participants was 394. The researcher received responses from 388 subjects.

### 2.2 Variables and Instruments

The following factors and tools were used in the study: academic performance, video gaming habits, and sociodemographic data. GPA served as the primary metric for assessing academic performance. School performance was evaluated through several questions that assessed different aspects of scholarly activities. The GPA was used as a primary outcome to measure scholastic performance. The GPAs were measured on a percentage scale and categorized into two categories. The categories were average academic performance (GPA= 60-89.9%) and high scholastic performance (GPA=90-100). The GPA cutoff point was selected to facilitate the comparison between high-achieving students (A students) vs. non-high-achieving students. Furthermore, this categorization was made after an initial screening of the data. This screening revealed a clear distinction in the measured outcomes between students who achieved a GPA of more than 90% and the rest of the students. Data regarding gaming behavior, such as the frequency of using gaming apps during classes, was collected using questions that assessed different aspects of gaming behavior. These questions were formulated based on the available literature related to the topic. The survey included additional questions that assessed the characteristics of gaming application use and the extent of their impact on academic performance.

Problematic gaming behaviour was assessed using a validated Arabic version of the Internet Gaming Disorder Scale–Short-Form (IGDS9-SF) [33]. The IGDS-9SF is a validated tool developed based on the nine-item criteria suggested by the Diagnostic and Statistical Manual of Mental Disorders, 5^th^ edition (DSM-5). The scale consists of 9 items, measured on a 5-point Likert scale, ranging from “Never” to “Very often.” The score on this scale ranges from 9 to a maximum of 45 points, with higher scores indicating a higher degree of internet gaming disorder. The IGDS9-SF does not directly quantify time spent gaming or frequency of play, but rather captures clinical symptoms associated with gaming disorder.

The impact of social media addiction on mental health was assessed using the validated Arabic versions of the Patient Health Questionnaire-9 (PHQ-9), Generalized Anxiety Disorder-7 (GAD-7) [34], and the Perceived Stress Scale (PSS) [35]. The tools are brief self-reported questionnaires that assess the presence of depressive anxiety and stress symptoms, respectively. The PHQ-9 is a self-reported version of the PRIME-MD diagnostic tool. It consists of 9 items measured on a 4-point Likert scale ranging from “not at all” to “nearly every day”. The maximum score in this tool is 27, and a cut-off point of more than 9 has been suggested to identify patients with depression [36-38]. The GAD-7 is a self-report tool and is also validated in Arabic [39, 40]. It consists of 7 items, each measured on a 4-point Likert scale. The scale ranges from “never” to “almost every day.” The maximum score in this tool is 21, with cutoff points of 5 and 10, and is used to identify mild, moderate, and severe anxiety, respectively [39, 40, 42]. The PSS is a self-reported questionnaire based on the psychological conceptualization of stress [38, 41, 42]. It is validated in Arabic among Jordanians [42]. It assesses the extent to which life events have been perceived as uncontrollable and unpredictable over the last month. The PSS consists of 10 items on a 5-point Likert scale ranging from “Never” to “Very often”. The maximum score of this tool is 40, with higher values indicating higher levels of stress [38, 41].

### 2.3 Statistical Analysis

Calculations were made for sociodemographic variables using descriptive statistics. Using χ^2^ for categorical data, the distribution of academic performance levels is stratified by these variables. A Student's t-test was used for variables measured as continuous data. The Statistical Package for Social Sciences (SPSS) version 24 (IBM SPSS Statistics for Windows, Version 24.0. Armonk, NY: IBM Corp.) was used for all statistical analyses. A p-value < 0.05 was the threshold for statistical significance.

## 3 RESULTS

In this study, the total number of participants was 388, with a mean age of 13.49 years (SD = 1.2). The mean age was 13.55 years for students with an average GPA and 13.45 years for those in the high GPA group. There were no significant differences between the two groups (p = 0.47). More than half of the respondents were male (59.5%). The majority of students (98.97%) lived with their families.

Regarding psychological measures, a t-test analysis was carried out to evaluate the association between psychological symptoms and GPA (Table **1**). The findings indicated that gaming behavior showed a significant association with GPA levels (p = 0.04), with students in the high GPA group and high gaming scores (Mean = 32.6, SD = 9.04), compared to students with an average GPA and gaming scale (Mean = 30.6, SD = 8.5). The depression scale also showed a significant association with GPA levels (p = 0.02), with students who have high GPAs reporting high levels of depression scale (Mean = 18.7, SD =6.1), compared to a group of students who have average GPA (Mean = 17.2, SD = 5.7). However, the anxiety and stress scores were not significantly associated with GPA. These results suggest a significant association between academic performance (as measured by GPA level) and the depression scale.

### 3.1 Predictors of Academic Performance

A binary logistic regression analysis identified and assessed the predictors of academic performance (high GPA) (Table **2**). The primary predictors evaluated were age, gaming scale, stress score, depression, and anxiety score. For high GPA, age (p = 0.46) and gender (p = 0.57) were not significant predictors, but depression was a significant predictor (OR 1.07; 95% CI: 1.01-1.13). Based on the results, the anxiety score showed a slight association (OR 0.95, CI: 0.9-1.01), but no statistically significant association with academic performance (p = 0.1). The gaming scale and stress were also not significant predictors of high academic performance.

### 3.2 Predictors of Depression Severity

The multiple linear regression analysis identified and assessed the predictors of depression severity (Table **3**). Five predictors were evaluated: age, gender, gaming scale, stress score, and anxiety score. This model was statistically significant (F = 94.25, p < 0.0001, Adjusted R^2^ = 0.55). The significant positive predictor was the anxiety score (β = 0.60, 95% CI: 0.54 – 0.71, p < 0.0001), indicating that a higher anxiety score was associated with increased depression severity. The gaming scale was also a significant positive predictor (β = 0.14, 95% CI: 0.043 – 0.15, p < 0.0001). In contrast, stress score was negatively associated with depression severity (β = -0.13, 95% CI: -0.19 – -0.04, p = 0.002), indicating that lower stress levels were related to higher depression severity. Other variables, including age and gender, were not significant predictors of depression severity.

### 3.3 Characteristics of Gaming Applications Usage and Depression Severity

Table **4** presents a comparative analysis of the characteristics of gaming application usage and depression severity. The number of daily gaming applications did not significantly differ between the different levels of depression severity (p = 0.75). A borderline significant association was observed between depression severity and the use of gaming applications during class (p = 0.08). The impact of gaming application usage on the ability to meet deadlines showed a significant difference between groups (p < 0.0001). Among students with severe depression, many reported that gaming frequently impacted their ability to meet deadlines. Similarly, there was a significant association between depression severity and the impact on exam preparation (p < 0.05). Those experiencing severe depression were more likely to claim that gaming hindered their exam preparation.

Another critical factor was the ability to attend the first lesson. Students with severe depression reported trouble getting out of bed and making it to class (p < 0.0001). Gaming significantly impaired students' ability to focus during class (p < 0.0001), with 63.9% of severely depressed students reporting concentration difficulties due to gaming. Regarding instructors asking students to stop gaming, 91.7% of severely depressed students reported that their instructor requested them to stop gaming during class (p < 0.0001). Furthermore, students with severe depression felt that gaming had a negative impact on their academic performance (p < 0.05).

## 4 DISCUSSION

This study offers important novel information about how gaming habits impact academic achievement, stress, anxiety, and depression. The findings revealed that gaming behavior had a negative impact on academic performance. However, a prior study by Dumrique and Castillo (2018) implied no clear association between gaming activity and educational achievement and GPA levels [43]. However, another study consistent with our findings, for example, Abbasi *et al.* (2021) [52] and Sharma and Pandey (2017), [47] suggested that the relationship between gaming and academic performance is intricate and might be affected by various factors, such as the type of games, duration of gaming, and individual differences.

Our findings reported that anxiety levels were not significantly correlated with GPA. However, anxiety remains a critical factor that can disrupt academic performance. Even in the absence of a direct correlation with GPA, the results suggest that anxiety can have a crucial negative impact on academic performance by interfering with concentration, motivation, and test-taking abilities. Previously published studies supported these findings [44-46]. Additionally, our findings reported that the relationship between stress and academic performance was consistent with previous research [47-49]. Although there was no significant correlation between stress levels and GPA, stress can still have a detrimental impact on academic performance and overall well-being. McCurdy *et al*. (2022) stated that stress acts as a motivator for achieving better grades. However, it was found that stress negatively impacts academic performance, likely due to higher levels of anxiety and depression resulting from various stress factors among high school students [50].

Moreover, our results also indicated that high-GPA students reported higher IGDS9-SF scores, which may seem like a counterintuitive result. These results suggest that high-GPA students may spend time in gaming as a form of psychological escape from the stress associated with their academic achievements. These students may also engage in structured gaming without it directly harming their academic performance, but still experience internalized stress that contributes to depressive symptoms. It is also possible that personality traits, such as perfectionism or higher self-awareness among high achievers, could contribute to both increased psychological distress and gaming behavior. These hypotheses were demonstrated by previous researchers who found that academic stress increased gaming disorder tendency mediated through escape and coping motives [51-54]. These findings suggest a potential pattern worth further investigation, *i.e.*, high-achieving students may experience greater mental health burdens alongside increased gaming behavior. It suggests that academic achievement may be associated with increased stress and pressure, which can contribute to mental health problems [55-57]. This aligns with the findings of a meta-analysis study demonstrating that although depression is associated with lower educational attainment, the association between depression and educational attainment in young people is unclear, and there is a clear need for mental health and educational support among children and adolescents with depression [58]. This underscores the need for early interventions, counseling, and mental health support systems within schools and universities to address the psychological burden of high academic achievement [48, 58]. Given that our study is a cross-sectional design, these associations should be interpreted cautiously and explored further through longitudinal or experimental designs.

In our study, anxiety was found to be a significant predictor of depression severity at all levels, from moderate to severe. Additionally, it was discovered that the IGDS9-SF, which measures the severity of problematic gaming behavior and was identified as a significant predictor of moderately severe and severe depression, also predicted both mild and severe depressive states. This indicates that students exhibiting more symptoms of disordered gaming, such as loss of control, withdrawal, or functional impairment, may be at greater risk for experiencing depressive symptoms.

Moreover, our results demonstrated an unexpected finding, *i.e.*, a negative association between perceived stress and depression severity in the multiple regression model. This contradicts much of the existing literature, which commonly shows a positive link between stress and depression. A possible explanation may involve conceptual overlaps between stress and anxiety scores, which can influence the direction and strength of coefficients in regression models [59, 60]. Additionally, individuals experiencing more severe depressive symptoms may report emotional numbness or disengagement, leading to lower perceived stress levels despite elevated depressive states. These possibilities underscore the complexity of interpreting cross-sectional psychological data and highlight the need for further research using mediation or longitudinal models.

The findings also demonstrated that excessive gaming may exacerbate depressive symptoms. This is consistent with the findings reported by Stavropoulos (2022), which demonstrated that the interaction between anxiety and problematic online gaming behaviors strongly influences the severity of depression. These results suggest that gamers experiencing anxiety are at a heightened risk of developing depression, with the severity of their symptoms worsening as gaming-related issues increase and lessening when problematic gaming behaviors are reduced [32]. Similarly, Yuan (2021) reported findings that gaming, particularly in individuals with pre-existing anxiety, can significantly contribute to depression severity [61].

Our results indicated that age is a significant predictor of academic performance. Older students had higher intermediate GPAs, suggesting that maturity, better study habits, time management skills, and experience contribute to academic success. This is consistent with findings from previous literature [62, 63].

The result of this study suggests a complex relationship between gaming applications and depression severity among students. The frequency of playing games during class was significantly associated with depression scores. These findings align with a prior study conducted by Wei *et al.* [64], which found that students with severe depression were more likely to engage in gaming during class, leading to negative academic consequences, such as difficulty attending class, meeting deadlines, and preparing for exams. These findings are consistent with other studies demonstrating that excessive gaming may have detrimental effects on mental health, especially in those who already have vulnerabilities. The results also imply that the context of gaming, for example, playing games in class, may exacerbate the detrimental impacts on mental health [64, 65].

Our findings highlight several important implications for educators. One important finding is that instructors' approach to requests to stop playing differs depending on the degree of depression. This means that teachers may be more likely to help children who are misbehaving or showing clear signs of struggling, but they may ignore others who may be dealing with depression privately. Moreover, our results revealed that gaming behavior and anxiety are significant predictors of depression severity. For children at risk of developing or worsening mental health problems, this underscores the value of early intervention and assistance. It is also recommended that future research could benefit from leveraging advanced computational tools for early mental health screening. For example, a recent study demonstrated how artificial intelligence (AI)-driven and advanced machine-learning techniques can enhance the accuracy and reliability of diagnosing mental disorders [66]. Similarly, the study by Ali *et al.* (2023) demonstrated that neural network models could be adapted for predicting mental health risk in children [67]. These studies offer new insights for future research, particularly those related to utilizing new technologies as a pathway for predictive modeling and personalized interventions. These interventions aim to integrate gaming behavior, emotional distress markers, and academic performance to identify at-risk youth early and intervene more effectively.

## LIMITATIONS

Although the study discovered a relationship between depression and academic achievement, it was unable to prove a cause-and-effect relationship due to its cross-sectional design. Future longitudinal studies are needed to investigate the specific variables, such as parental involvement, self-regulation skills, and gaming motivation, that influence the relationship between gaming and academic achievement. While the present study’s sample included students from both public and private schools, the generalizability of the results may be limited by sample size and participant characteristics. Potential socioeconomic bias also cannot be entirely ruled out. Future studies should aim for broader representation across various geographic regions and socioeconomic backgrounds in Jordan. Additionally, the binary categorization of GPA (“average” vs. “high”) may not fully capture the variation in academic performance. However, this binary classification may oversimplify outcomes. Future studies should consider analyzing GPA as a continuous variable or applying more detailed performance groupings to gain a deeper understanding of the relationship between GPA and academic performance. Moreover, several potential confounding variables were not measured in this study, including socioeconomic status, non-gaming screen time, sleep quality, and family dynamics. These factors may independently influence gaming behavior, mental health, and academic outcomes. The absence of these variables limits our ability to isolate the specific effects of gaming. Incorporating these confounders into future research will enable a more accurate understanding of the complex interplay between psychological well-being, academic success, and digital behavior. Finally, researchers should explore the mechanisms underlying the observed associations, such as social isolation, cognitive impairment, or sleep disruption, to better understand how gaming may influence depression. Investigating the role of gaming applications and the context in which they are used may also offer critical insight into the risk factors of gaming-related mental health outcomes.

## CONCLUSION

This study provides important insights into the complex relationship between mental health and academic achievement, particularly in relation to the use of video games. According to research, social isolation and academic pressure may put high-GPA achievers at greater risk of developing depression. Additionally, this study explores the factors that predict the severity of depression. While the IGDS9-SF scores were associated with severe and moderately severe depression, anxiety was found to be a consistent predictor across all levels. These findings highlight how important it is to manage anxiety as well as any other gaming-related issues to avoid and treat depression. The findings of this study have important implications for both mental health practitioners and educational institutions. Schools should consider establishing rules and initiatives to promote responsible gaming and provide support for children with mental health issues.

## AUTHORS’ CONTRIBUTIONS

The authors confirm their contribution to the paper as follows: S.R.: Study concept or design; K.H.A: Data analysis or interpretation; R.M.: Writing the paper: I.B.: Writing, reviewing, and editing; L.T.A.: Methodology: B.M.: Data collection: A.A.: Data curation. All authors reviewed the results and approved the final version of the manuscript.

## Figures and Tables

**Table 1:** Baseline attributes and GPA levels of participants.

Population Characteristics		Total	GPA Level		p-value	
			Average	High		
Age (y) (SD)		13.49 (1.2)	13.55	13.45	0.47	
Gender N (%)	Male	231 (59.5)	76 (58.4)	155 (60.1)	0.83	
	Female	157 (40.5)	54 (41.6)	103 (40.9)		
Accommodation N (%)	With Family	384 (98.97)	129 (99.23)	255 (98.84)	1	
	Dorm	4 (1.03)	1 (0.77)	3 (1.16)		
Gaming Scale, Mean (SD)		31.8 (8.9)	30.6 (8.5)	32.6 (9.04)	0.04	
Depression Scale, Mean (SD)		18.6 (6)	17.2 (5.7)	18.7 (6.1)	0.02	
Anxiety Score, Mean (SD)		12.6 (5.8)	12.3 (5.8)	12.7 (5.7)	0.47	
Stress Score, Mean (SD)		17.9 (6.5)	18.3 (6)	17.8 (6.7)	0.5	

**Table 2:** Binary logistic regression model for predictors of high GPA levels.

	**OR**	**95% CI**	**p-value**
**Age**	0.93	0.78-1.13	0.46
**Gender**	0.87	0.54-1.41	0.57
**Depression**	1.07	1.01-1.13	0.02
**Anxiety Score**	0.95	**0.9-1.01**	0.1
**Gaming Scale**	1.02	**0.99-1.05**	0.12
**Stress Score**	1	**0.97-1.05**	0.75

**Table 3:** Standardized regression analysis for predictors of depression.

	**Standardized Coefficients Beta**	**95% CI**	**p-value**
**Age**	-0.01	-0.43-0.31	0.76
**Gender**	0.05	-0.34-1.49	0.22
**Anxiety score**	0.6	0.54-0.71	0.0001
**Gaming Scale**	0.14	0.043-0.15	0.0001
**Stress Score**	-0.13	-0.19-(-0.04)	0.002

Adjusted R^2^=0.55; F=94.25; p=0.0001

**Table 4:** Characteristics of gaming application usage and depression severity.

**Items**	**Depression Severity**					**P-value**
	**Normal**	**Mild**	**Moderate**	**Moderately severe**	**Severe**	
**Number of Gaming Applications Used Daily, N (%)**						
**1**	3 (25)	5 (17.2)	14 (18.2)	23 (18.1)	26 (18.1)	**0.75**
**2**	2 (16.7)	2 (6.9)	13 (16.9)	22 (17.5)	29 (20.1)	
**3**	2(16.7)	5 (17.5)	15 (19.5)	26 (20.6)	35 (24.3)	
**4**	1 (8.3)	6 (20.7)	13 (16.9)	19 (15.1)	17 (11.8)	
**5**	3 (25)	3 (10.3)	3 (3.9)	13 (10.3)	12 (8.3)	
**More than 5**	1 (8.3)	8 (27.6)	19 (24.7)	23 (18.3)	25 (17.4)	
**Use of Gaming Applications During Class, N (%)**						
**Never**	7 (58.3)	17 (58.6)	**56 (72.7)**	**96 (76.2)**	**121 (84)**	**0.08**
**Rarely**	2 (16.7)	5 (17.2)	9 (11.7)	12 (9.5)	12 (8.3)	
**Sometimes**	0 (0)	3 (10.3)	9 (11.7)	9 (7.1)	5 (3.5)	
**Often**	1 (9.3)	2 (6.9)	2 (2.6)	3 (2.4)	3 (2.1)	
**Always**	2 (16.7)	2 (6.9)	1 (1.3)	6 (4.8)	3 (2.1)	
**Impact of Using Gaming Applications on Students' Ability to Meet Deadlines, N (%)**						
**Never**	5 (41.7)	7 (24.1)	**27 (35.1)**	**50 (39.7)**	**78 (54.2)**	**0.0001**
**Rarely**	2 (16.7)	6 (20.7)	14 (18.2)	33 (26.2)	21 (14.6)	
**Sometimes**	3 (25)	7 (24.1)	24 (31.2)	32 (25.4)	21 (14.6)	
**Often**	2 (16.7)	5 (17.2)	9 (11.7)	8 (6.3)	4 (2.8)	
**Always**	0 (0)	4 (13.8)	3 (3.9)	3 (2.4)	2 (1.4)	
**Impact of Using Gaming Applications on Students' Exam Preparation, N (%)**						
**Never**	6 (50)	10 (34.5)	**22 (28.6)**	**53 (42.1)**	**81 (56.3)**	**0.008**
**Rarely**	2 (16.7)	6 (20.7)	15 (19.5)	26 (20.6)	30 (20.8)	
**Sometimes**	3 (25)	6 (20.7)	27 (35.1)	27 (21.4)	25 (17.4)	
**Often**	1 (8.3)	4 (13.8)	8 (10.4)	15 (11.9)	8 (5.6)	
**Always**	0 (0)	3 (10.3)	5 (6.5)	5 (4)	0 (0)	
**Effect of Gaming Apps on Students' Ability to Get Up and Go to First Class the Following Day, N (%)**						
**Never**	7 (58.3)	8 (27.6)	**25 (32.5)**	**67 (53.2)**	**96 (66.7)**	**0.0001**
**Rarely**	2 (16.7)	4 (13.8)	21 (27.3)	28 (22.2)	22 (15.3)	
**Sometimes**	0 (0)	7 (24.1)	17 (22.1)	17 (13.5)	12 (8.3)	
**Often**	1 (8.3)	5 (17.2)	9 (11.7)	6 (4.8)	9 (6.3)	
**Always**	2 (16.7)	5 (17.2)	5 (6.5)	8 (6.3)	5 (3.5)	
**Impact of Using Gaming Applications on Students' Ability to Focus on Class, N (%)**						
**Never**	6 (50)	10 (34.5)	**27 (35.1)**	**69 (54.8)**	**92 (63.9)**	**0.0001**
**Rarely**	2 (16.7)	4 (13.8)	20 (26)	31 (24.6)	34 (23.6)	
**Sometimes**	1 (8.3)	7 (27.1)	21 (27.3)	15 (11.9)	8 (5.6)	
**Often**	1 (8.3)	4 (13.8)	6 (7.8)	5 (4)	8 (8.6)	
**Always**	2 (16.7)	4 (13.8)	3 (3.9)	6 (4.8)	2 (1.2)	
**Frequency of Instructors Asking Students to Stop Using Electronic Games During Lectures or Practical Sessions, N (%)**						
**Never**	9 (75)	18 (62.1)	**59 (76.9)**	**11 (88.1)**	**132 (91.7)**	**0.0001**
**Rarely**	1 (8.3)	6 (20.7)	9 (11.7)	8 (6.3)	6 (6.2)	
**Sometimes**	2 (16.7)	2 (6.9)	7 (7.1)	7 (5.6)	5 (3.5)	
**Often**	0 (0)	2 (6.9)	0 (0)	0 (0)	1 (0.7)	
**Always**	0 (0)	1 (3.4)	2 (2.6)	0 (0)	0 (0)	
**Students' Perception of the Negative Impact of Using Gaming Applications, N (%)**						
**Never**	2 (16.7)	6 (20.7)	**17 (22.1)**	**36 (28.6)**	**56 (38.9)**	**0.001**
**Rarely**	5 (41.7)	4 (13.8)	21 (27.3)	27 (21.4)	42 (29.2)	
**Sometimes**	2 (16.7)	6 (20.7)	28 (36.4))	36 (28.6)	29 (20.1)	
**Often**	1 (8.3)	8 (26.7)	6 (7.8)	22 (17.5)	13 (9)	
**Always**	2 (16.7)	5 (17.2)	5 (6.5)	5 (4)	4 (2.8)	

## Data Availability

Data will be available upon request via email to the corresponding author.

## STANDARDS OF REPORTING

STROBE guidelines were followed.
